# Nurses’ and care workers’ experiences of spiritual needs in residents with dementia in nursing homes: a qualitative study

**DOI:** 10.1186/1472-6955-13-12

**Published:** 2014-04-15

**Authors:** Liv Ødbehr, Kari Kvigne, Solveig Hauge, Lars Johan Danbolt

**Affiliations:** 1 Department of Nursing and Mental Health, Faculty of Public Health, Hedmark University College, P.O Box 400, N- 2418, Elverum, Norway; 2 Institute of Health and Society, Department of Nursing Science, University of Oslo, P.O. Box 1130 Blindern, N- 0318 Oslo, Norway; 3 Institute of Nurse Education, Nesna University College, N- 8700 Nesna, Norway; 4 Institute of Health Sciences, Faculty of Health and Social Studies, Telemark University College, N- 3901 Porsgrunn, Norway; 5 Centre of Caring Research-Sothern Norway, Institute of Health Sciences, Faculty of Health and Social Studies, Telemark University College, N- 3901 Porsgrunn, Norway; 6 Center for psychology and religion, Innlandet Hospital Trust, P.O. Box 68, N- 2312, Ottestad, Norway; 7 Oslo School of Theology, P.O. Box 1153, Blindern N- 0318 Oslo, Norway

**Keywords:** Spiritual needs, Dementia, Nursing home, Phenomenological hermeneutics, Nursing care

## Abstract

**Background:**

The aim of the study was to investigate nurses’ and care workers’ experiences of spiritual needs among residents with dementia in nursing homes. Nurses claim to practice holistic nursing. Nevertheless, there is little knowledge about how to recognise spiritual needs in residents with dementia.

**Methods:**

The study was conducted using a qualitative method with an exploratory design. Eight focus- group interviews in four Norwegian nursing homes were performed from June 2011 – Jan 2012. Using open-ended research questions, a total of 31 participants were asked to share their understanding and experiences regarding residents’ spiritual needs. The interviews were analysed using a phenomenological – hermeneutical method.

**Results:**

The nurses’ and care workers’ experiences of residents’ spiritual needs were related to three main themes; i) The need for serenity and inner peace, described as “*contemplative and restful moments*” and “*calmness due to familiarity*”, *ii*) The need for confirmation, described as “*love and proximity*” and *iii*) The need to express faith and beliefs, described as “*participate in worship and prayers*” and “*approaching death*”.

The comprehensive analyses revealed that the nurses believe the residents’ spiritual needs were linked to the residents’ previous sources of finding meaning, in relation to inter-personal, intra-personal and trans-personal dimensions in residents’ lives.

**Conclusions:**

Nurses' and care workers’ experiences of spiritual needs in people with dementia are very similar to the findings for the general population regardless of the severity of the dementia. The study’s relevance to clinical practice indicates the importance of developing more knowledge about how people with dementia in nursing homes express spiritual needs and how to observe and interpret such needs.

## Background

Holistic nursing targets the physical, mental, social and spiritual needs in people’s lives [1,2]. Spiritual needs are particularly important in crisis-like periods and at the end of life [3-5]. Dementia is characterised by impaired memory and control of behaviour and emotions [6], and dementia can cause a crisis-like state involving mental and spiritual pain and suffering [7-9], such as loneliness, grief and fear. Although nurses claim that they work from a holistic nursing perspective, they admit to a lack of knowledge about how to meet residents’ spiritual needs in general [10-14], but especially for people with dementia [15,16].

Research regarding care workers’ experience of residents’ spiritual needs in nursing homes is scarce. A search of the CINAHL, AgeLine, Medline and PsychInfo databases was performed initially in October 2012. We used the following search terms: dementia AND spiritual needs AND nursing homes. We limited to the search to articles that were peer-reviewed, in the English language, published 2007 – 2012 and included participants’ age 65 and older. The search resulted in 168 articles in which only one empirical article focused on nurses’ experiences of residents’ spiritual needs in nursing homes [17]. In Bursell and Mayers’ [17] phenomenological study, eleven inter-professional health workers were interviewed to understand their perspective of residents’ spirituality and spiritual needs. The study revealed that the health workers thought that spiritual needs were linked to faith in God, to being acknowledged, providing quiet time and space, listening to music or other activities, such as smelling flowers.

However, there were a few studies that described spiritual needs from the residents’ perspective. The findings revealed that residents identified spiritual needs as experiences of meaning in different ways. The sense of meaning could be provided by relationships with family members, friends or through communication [18,19]. Residents drew on several sources of meaning and emphasised the need of belonging and the need for cognitive and psychological capability [19]. One of the studies showed how the elderly transcend in later life [20]. The study described the experiences of meaning as to feel valued by oneself and others, to be loved and be able to maintain the memory of love as well as belief in God or a higher power. How nurses and care workers recognise and experience these spiritual needs by not asking the residents openly is uncertain, particularly if the residents’ ability to verbally express their own needs is reduced. *The aim* of this study was therefore to investigate nurses’ and care workers’ experience of spiritual needs in residents with dementia in nursing homes.

### Theoretical perspective

Spiritual needs have traditionally been understood in terms of trust, forgiveness, love, peace, hope and purpose [10,21-26]. Several studies also refer to spiritual needs as connectedness to God, others, self and whatever the person values [27,28]. It reveals a basic human need for belonging and relatedness to dimensions that create meaning, which is highlighted in the definition of spirituality by Reed [27]:

“Spirituality refers to the propensity to make meaning through a sense of relatedness to dimensions that transcend them self in such a way that empowers and does not devalue the individual. This relatedness may be experienced intra-personally (as a connectedness within self), inter-personally (in the context of others and the natural environment) and trans-personally (referring to a sense of relatedness to the unseen, God, or power greater than the self and ordinary source).

Reed [27] emphasises a human’s search for meaning is essential and is recognised in several other studies that describe spiritual needs [29-33]. The types of dimensions or sources humans rely on to make meaning intra-personally, inter-personally and trans-personally are still unclear. In Schnell’s [34] study, four main sources related to creating meaning were highlighted:

(1) *Self*- *transcendence*, (1a) *Vertical self*-*transcendence*: orientating towards an immaterial, supernatural power such as faith in God, and (1b) *Horizontal self*- *transcendence*: taking responsibility for (worldly) affairs beyond one’s immediate concerns, such as self-knowledge or unison with nature.

(2) *Self*- *actualisation*, employing, challenging and fostering one’s capacities, such as experiences of freedom, creativity, power.

(3) *Order*, *holding on to values*, *practicality*, *decency*, *and the tried and tested*, which can be linked to moral and traditions.

(4) *Well*-*being and relatedness*, cultivating and enjoying life’s pleasures in privacy and company, such as experiences of harmony, comfort, love, fun, community.

Despite the different descriptions, we regarded Reed’s [27] definition of spirituality as a inter-personal, intra-personal and trans-personal quest for meaning that is consistent with Schnell’s [34] perspective on making meaning. People use a variety of sources to create meaning individually and reveal the importance of valued spiritual needs in their life [35]. In accordance with the understanding of spirituality according to Reed’s [27] definition of inter-personal, intra-personal and trans-personal quest for meaning and Schnell’s [34] sources of meaning, we sought to understand the nurses’ experiences of residents’ spiritual needs based on observations and reflections.

## Methods

### Study design

To investigate professionals’ experiences in a scarcely studied field, using a qualitative method with an exploratory design was appropriate. The advantage of focus groups is that the group is jointly creating data through the participants’ sharing of similar experiences [36-38].

### Setting

Four nursing homes participated in the study. All four nursing homes had special expertise in caring for residents with dementia by employing competence-raising measures for nurses and care workers for several years. Studies conducted in a Nordic context reveal that 81% of residents in Norwegian nursing homes have dementia and are in need of extensive help throughout the day [39]. The nursing homes in this study were located in southern Norway. The nursing homes had the following characteristics: Nursing home A consisted of 216 employees (51 registered nurses (RNs), 129 care workers, and 36 assistants) and included four departments with a total of 103 residents; Nursing home B consisted of 207 employees (48 RNs and 68 care workers and 91 assistants/other) and five departments which included a total of 116 residents; Nursing home C consisted of 74 employees (20 RNs and 32 care workers and 22 assistants/other) and included four departments and a total of 29 residents; Nursing home D consisted of 70 employees (30 RNs and 40 care workers) and three departments with a total of 54 residents. The total number of employees who were not ethnically Norwegian was 44, and they all were fluent in the Norwegian language. Ethnicities included Hispanic, Polish, Dutch, Swedish and African nurses or care workers.

### Participants

Purposive sampling was used to select participants, and information was given orally and in written form to the head nurses, who chose and requested two participants from their department to interview. Participants had to be permanent employees in the nursing home and have worked more than one year in dementia care. Because there were more care workers than nurses, we invited both professions to participate in the study. A total of 30 women and 1 man were included, fifteen nurses and sixteen care workers, and four of them were educated in palliative care, mental health and geriatric nursing. Four of the participants were under 30 years of age, one between 30 – 50 years, and sixteen of the participants were older than 50 years. The participants’ working experience included the following: <5 years (n = 4), between 5–10 years (n = 10) or more than 10 years (n = 17). Twenty six nurses and care workers from the four institutions participated in the first interview. In the follow-up interview, twenty participated. We preferred that the same professionals participated in both interviews, but because of different shifts, five new participants were included in two of the follow-up interviews.

### Data collection

Data were collected from June 2011 to January 2012. At least one interview was performed at each of the four institutions, and a follow-up interview was conducted. Research shows that recurrent knowledge dialogue in a particular group may increase the understanding of a theme [40,41]. Through having a follow-up interview, we wanted to obtain the participants' reflections after the initial interview and deepen some of the topics that the nurses discussed in the first interview [40]. The same moderator (first author) and observer (second author) conducted all eight interviews that were located in the nursing homes, lasted 1 ½ - 2 hours and recorded on audio files. Time from the first interview to the follow-up interview was approximately six weeks. The number of group members ranged from 4–8. All participants were given a written topic guide with open questions, and sample questions included: "*Is spiritual care a familiar term in care for people with dementia*?” "*In what ways do* residents *express spiritual needs*"? “*What knowledge among nurses is of importance in the provision of spiritual care in practice*”? The moderator asked follow-up questions, and the observer asked supplementary questions [42]. All interviews were transcribed by the first author. The eight interviews generated 228 pages of text, with line spacing of 1.5. The first interview was transcribed before the follow-up interview to question the data. A summary of the first interview was presented initially at the follow-up interview to maintain continuity. The researchers intended to receive comments or corrections. After eight interviews, we experienced saturation and ended the interviewing.

### Analysis

We applied Lindseth and Nordberg’s [43] phenomenological-hermeneutical method for interpreting interview text, because the aim of the method was to disclose the meaning of nurses’ experience of residents’ spiritual needs [44]. The method of analysis was inspired by Ricoeur’s philosophy [45]. Interpretations of the text consist of a dialectic movement between understanding the whole text and parts of the text, which is consistent with the hermeneutic method [46]. This closeness and distance of the text implies interpreting the text in terms of reading the text for what it says and further understanding what the text suggests. The analysis followed three steps: naïve reading, structural analysis and formulation of a comprehensive understanding.

#### *Naïve reading (initial reading)*

The text was read several times to grasp the meaning as a whole. During the reading, we tried to focus on the nurses’ lived experiences as they reflected on the residents spiritual and existential expressions. Naïve reading was discussed between the researchers and further guided the thematic structural analysis.

#### *Structural analysis*

All four researchers conducted data coding. First, the text was divided into meaning units. We reflected on the meaning units based on the background of the naïve understanding and then condensed the units to reflect the essential meaning. We read through all of the condensed meaning units and reflected on their similarities and differences. Sub-themes were then created, which were assembled to themes and main themes. We further reflected on the themes in relation to the naïve understanding, and if we discovered a discrepancy between the naïve understanding and themes, the structural analysis process was repeated until there was compliance.

#### *Comprehensive understanding*

We reflected on the themes and sub-themes in relation to our pre-understanding, research question, and the context of the study, in which we sought a comprehensive understanding. The credibility of the findings was assessed in the process of coding, in that we selected significant sections from the participants’ statements and identified explicit themes. We sought to safeguard transparency and trustworthiness through quotations from different participations in the presentation of the findings. During the entire process, we attempted to assess consistency between the data presented and the study findings, including both major and minor themes. By comparing themes to the naive reading, we strengthened the validity of the analysis.

### Ethical considerations

Consistent with Norwegian legislation, collecting data about professional healthcare workers job experiences has to be assessed ethically by Data Protection Official for Research at the Norwegian Social Science Data Services (NSD). This was done in the current study in April 2011 (with reference number 26783). NSD confirmed that the study met the requirements for ethical soundness in relation to standards and codes of ethics. The study was also performed according to the Declaration of Helsinki. The licenses of leaders from the municipalities and the nursing institutions were obtained, and participation was voluntary. To ensure the participants' well-being, integrity and dignity throughout the interview process, the importance of safeguarding the confidentiality of each participant was emphasised. Written informed consent was obtained from all participants, and all attendees were informed that they could withdraw from the study at any time.

## Results

In the results, we use the term “nurses” to simplify the presentation, and the care workers are therefore included in the term. The quotes are presented indicating the specific interview (specified in capital letters), which of the informants that spoke in the group (indicated by numbers) and the page number in the transcribed text.

### Naive reading

Nurses understood the residents’ restlessness and expressions of negative feelings, including anxiety, fear, despair, loneliness, and confusion, which they interpreted as uncovered spiritual needs. They also noticed that some residents could verbally express demands for religious activities, such as prayer and singing hymns. Furthermore, they observed that residents wanted to connect to them on a personal level. The nurses described residents’ previous interests, such as nature experiences, culture and traditions as spiritual needs, as they saw they gave a sense of meaning to the residents.

### Structural analysis

The nurses’ and care workers’ experiences of spiritual needs in residents with dementia in nursing homes embodied three themes in the structural analysis: i) *The need for serenity and inner peace*, *ii*) *The need for confirmation*, and *iii*) *The need to express faith and beliefs*. Sub-themes are presented under each main theme with detailed descriptions.

### The need for serenity and inner peace

In spiritual experiences, the nurses realised something transgressed in the form of a peaceful moment. These experiences focused nurses’ attention on important values in residents’ lives.

Nurses observed that the dementia caused confusion and uncertainty for the residents.

They also experienced that the residents’ changed between a state of passivity or apathy and a state of inappropriate restlessness. Restlessness was interpreted as an expression of discomfort or lack of inner peace, and the nurses wanted to help the resident into a state of rest.

#### *Contemplative and restful moments*

The nurses believed that music could be a tool to reach residents’ spiritual needs and also to those residents who were apathetic. The music created a good atmosphere, and the residents’ reactions were generally seen as positive. In some cases, music affected residents’ emotions and evoked reactions in the form of tears, frustration or anger. One of the participants regarded such reactions as valuable expressions of spiritual needs that should not be prevented. One nurse said:

2 H: “Somebody sang Evert Taube, - and the tears started streaming, - and they said “look how distressed she is”. But I didn’t think it was distressing. It showed that she is a whole person. The music touched some of her memories, and maybe they were painful, - but I think that's OK. It makes you feel like a whole person, because that’s what we are”. (p 19)

Music was also used in addition to physical massage of residents’ feet, arms or shoulders. Nurses observed that the impact of such contact resulted in what the nurses interpreted as inner harmony of the resident. They saw that the resident was able to relax in a moment and feel a form of serenity. One nurse articulated about her experience:

3 J: “I found one CD with religious music way back in the patient’s drawer. So I put on the CD and gave her a gentle massage. As she sat there, the tears started rolling. She talks a lot, but struggles to communicate, so it’s difficult to know what she wants. But obviously, this music must have struck a chord in her, -it seemed very important to her. And she became very calm and balanced for a little while. So this was a good moment”. (p 1)

The residents sometimes expressed fear as to whether they could trust the nurses or not. Trust between nurses and residents’ were seen as essential and was created through an atmosphere that the nurses regarded as safe and kind.

#### *Calmness due to familiarity*

Many restless residents became tired during the day. Nevertheless, the nurses found that the residents could not manage to sit still, and the nurses believed they should not force them to be still. A better way to meet the residents’ needs was to engage them in familiar activities as a way to deflect the non-focussed wandering in the department. The nurses' observed that residents benefited from activities that were adapted to them individually. The nurses believed that when the residents were introduced to familiar activities, they most often responded positively by a sense of relief and calmness. One nurse exemplified this reaction in the following:

A 6: “A very restless resident couldn’t sit still during group gatherings, no matter what we did. He used to work outdoors a lot. So we took him for a walk and let him push a wheelbarrow. And in some way or another, I think this activity made sense to him”. (p 13)

Appropriate activities included walking in the garden, singing songs, talking about old objects and/or pictures, and listening to familiar poems or stories. The purpose of the activities was the residents’ experience of coping with their restlessness and to become settled. The resident’s altered behaviour was interpreted as the meaning in life was met.

### The need for confirmation

The nurses perceived that knowledge of residents’ background and life history was vital to understand the residents’ spiritual needs. Many patients struggled with what the nurses regarded as a sense of inferiority because they felt they were not accepted by the others. The nurses observed that vital values in residents’ lives were revealed in relationships, and these connections enabled the nurses to confirm the resident’s identity and to foster a sense of self-awareness. Nurses underlined that the residents also wanted to be identified for who they once were and in that way maintain a sense of self-worth and coherence. Similarly, confirming self-experiences might contribute to a feeling of belonging with the fellow people in the nursing home.

#### *Love and proximity*

The nurses thought that they may be the most important persons in the residents’ lives, and they perceived this role as a significant responsibility. The nurses observed that some residents were socially isolated, and therefore the nurses emphasised the importance of establishing an atmosphere of tenderness and love. Many residents enjoyed being escorted around, receiving a hug, holding hands, or just being near the nurses. One nurse expressed this closeness this way:

A 2: “They do not have to feel that they have lost themselves completely, but that they still are a full-fledged human being. We can try to maintain and enhance the patient’s interests and preserve what is left. Then, they can feel like a whole person and not just one that loses more and more of “self” and “disappears”. (p 10)

The nurses felt that the residents had great needs for social contact, both through physical proximity or just be with the residents. Although the social contact was mainly perceived by the nurses as positive and good, they also talked about the connection as exhausting in situations in which residents “clung” to them. One nurse stated:

1 D: “When we sit, the residents sit, and when we walk in the corridor, the residents follow us, it's like carrying a backpack”. (p 11)

The nurses observed that residents related to relatives and other fellow residents and that the social relationships were important to them. Some of the residents expressed thoughts such as: “*I am just stupid*” or “*I am totally alone*”. The nurses found that closeness and love were basic spiritual needs that were important to consider when residents struggled with low self-esteem and felt lonely.

### The need to express faith and beliefs

The nurses considered faith and beliefs to be important aspects of the residents’ lives. Nevertheless, these components were modestly discussed among the nurses. Commonly, the priest came and maintained worship in the nursing homes once a week and the nurses felt that this was helpful and that they could rely on the priest.

#### *Participate in worship and prayers*

The nurses considered the priest as a support in situations they did not know how to handle.

They observed that residents expressed solemnity in the worship and that most residents wanted to attend. One nurse explained:

1 E: “Even though they don’t consider themselves very religious, - showing respect for the priest and the church service is deeply ingrained in them”. (p 12)

The nurses experienced that singing familiar hymns was of great significance to the residents. The nurses explained that residents knew the hymns and they wanted to sing. Although their memory was reduced, they remembered the hymns.

D 4: I was called for to a lady who was very upset. I knew she was a Christian, so I asked if she wanted to sing a special song. And she did, - so we sang and she became quiet and fell asleep”. (p 3)

The nurses reflect on religious activities related to faith and beliefs. The nurses found that some residents stated that they wanted religious acts in the form of prayer. The nurses helped the residents to express their faith, and one nurse with an immigrant background said:

2 D: “A very religious lady asked me if we could say a prayer before she went to bed. So I asked her, “-you mean like say “The Lord’s prayer”, -right?” I told her I only knew it in Polish. “Yes, but can you fold your hands?” she asked. So we folded our hands, and I said that she could pray in Norwegian and I would do it silently in Polish. And that was OK with her. So that was a way to do it together, - a nice experience”. (p 2)

Sometimes residents expressed their needs more indirectly through body language in the form of gesticulation. In such cases, background knowledge of the resident was useful. The nurses said that when they noticed religious symbols or a Bible in the room, they tried to support the residents according to what they understood was the resident’s values and beliefs.

#### *Approaching death*

The nurses thought that spiritual needs affected all aspects of the residents’ life and that these needs were specifically visible in the care at the end of life. Many residents wanted to talk about death, but the nurses experienced that only few residents expressed that they were directly afraid of dying. Some residents claimed that their strongest fear was the fear of being alone and not the fear of death itself. For some residents, the discouragement was so intrusive that death was seen as an escape from the meaninglessness. One nurse told about an experience with a resident:

1 H: “The lady said: “Am I still alive today”? And I said yes you still are. She said she was tired of living. And I answered that I could understand that. And then she said: “*Oh*, *finally somebody who understands me. People say you*’*re doing fine*, *and now we*’*re going to do this and that*”. I think, - it’s important to take the patients seriously. They are tired, but they don’t need to be scared of dying. And that makes it easier for us if they tell us they want to die …”. (p 30)

The findings reveal that nurses related residents’ spiritual needs to both inner experiences and the outer relational life. They emphasised a broad understanding of residents’ spiritual needs, and the faith dimension was prominent.

## Discussion

The aim of this study was to investigate nurses’ and care workers’ experience of spiritual needs in residents with dementia in nursing homes. The nurses’ understanding of spiritual needs in residents with dementia was mainly based on the residents’ non- verbal, and to some extent, verbal expressions. Background knowledge of the residents’ lives and issues related to values was important to interpret the residents' expressions. The comprehensive understanding and reflections were based on the main findings: spiritual needs experienced inter- and intra-personally and spiritual needs experienced trans-personally.

### Spiritual needs experienced inter- and intra-personally

The nurses’ experience of residents’ relatedness to dimensions experienced inter-personally and/or intra-personally [27] overlapped and could not be easily separated, and the nurses did not separate these experiences. Nurses found residents’ spiritual needs of trust and love were exposed in social relationships and that the need to belong increased in residents’ because their lives were increasingly fragmented. To “belong” may strengthen residents’ self- esteem and reduce experiences of isolation and insecurity [10,47,48]. Nevertheless, the nurses did not ask the residents openly in what way they experienced a sense of belonging or not, mostly because they did not think that the residents could answer properly. Still, our study revealed that residents with dementia do not differ greatly from cognitively healthy people regarding spiritual needs of belonging, self- esteem and security [27].

The findings in this study also revealed that the nurses’ recognition of the residents’ need for inner peace could be achieved through involvement in familiar activities [17]. Walking in the garden and memory work strengthened experiences of recognition and relatedness [34] and were an integral part of residents’ overall well-being [26]. Nevertheless, the nurses in the current study seemed to consider being content in life as more important than to what extent the activities were relevant for the residents. The resident’s everyday life could therefore be easily overridden by too many activities. Additionally, residents with dementia managed in a limited extent to express their own needs. The nurses admitted it was difficult to assess the residents’ own experience of meaning by observing body language. The nurses also did not know how residents used to interpret meaning in life or the individual resident’s process of meaning making. Studies related to coping and creating meaning emphasise this issue but provide few answers [5,49-52]. The findings in this study revealed the nurses only rarely considered the deprivation of sources of meaning in people with dementia. The nurses did not reflect on the extent the activities were individualised or experienced as significant for the resident, in terms of meeting spiritual needs. When residents enter a nursing home, they often lose access to sources that used to be important to them and enriched their lives. However, these sources may be most important to maintain during this phase of life.

Nurses were challenged by retrieving the residents’ previous sources of meaning instead of ignoring them, but this obstacle was not greatly discussed by the nurses. They also did not discuss how ignoring residents’previous sources of meaning contributed to decreasing the resident’s experience of self-awareness and coherence [34]. Social interactions maintained a sense of coherence between the past and present in residents’ lives [15,16,53]. When the interactions were experienced as meaningful, the residents were transcended and the experiences empowered the individual [27]. Many of the residents in this study suffered from poor self-image and were in need of confirmation regarding their “own self”. For residents with dementia in nursing homes, the possibility for self-actualisation and freedom is generally weakened by the character of the disease [54]. Nurses in this study understood the importance of relationships and that the residents were dependent on them, but the nurses had little knowledge of “meaning making processes” that could be helpful in the care of the residents.

### Spiritual needs experienced trans-personally

Many of the nurses in this study were unsure whether they could address the resident’s need of faith in God in an appropriate manner. The nurses’ approach to the resident’s faith was trapped between their own attitudes and prejudices and a more traditional and accepted form of religious practice in which the priest played a central role. The nurses addressed the residents’faith in God in the form of self-transcendence [20,34] and trans-personal experiences [27]. The sources of meaning that benefited the residents were mostly related to supporting the resident’s expressions of faith by using resources available in the resident’s environment [27,34]. The nurses argued that it was important for the residents to live according to core values including trust, love, hope and peace [17,20], therefore proving they used "order" as a source of meaning making [34]. Research shows that faith in God is important for people and a central component in their lives [55]. Nevertheless, the nurses in the current study felt slightly uncomfortable about openly discussing religious questions with the residents. The nurses occasionally participated in the residents’ religious faith expressions. This type of spiritual need was mainly addressed by the priest.

Another spiritual need that was not greatly discussed was questions regarding death. Nurses observed that residents was not afraid of death but of loneliness or dying alone. The residents’ questions about death were not widely discussed by the nurses and the reason they did not talk with the residents about the last phase of life was unclear. The residents’ loneliness was not acknowledged, although research shows that this factor is a major problem among many fragile elderly [56]. The nurses considered faith-related matters to be important in residents’ lives. The nurses’ emphasis of spiritual needs related to experiences of meaning on different levels in life is important [50,57]. Generally, the spiritual needs of people with dementia were more similar than different to the rest of the population, aside from the limitations of dementia.

### Methodological considerations

The use of the phenomenological-hermeneutical method in focus group interviews has been questioned [58]. The methodological assumptions may challenge the use of group interviews and the researcher’s pre- understanding, which we have reflected on. First, the nature of phenomenology does not require individual interviews, although focus groups will allow for individual perspectives [36]. Second, the phenomenon is examined from several different aspects in a group interview, which phenomenology encourages [59]. Focus groups can therefore contribute to a broader understanding of the phenomenon and bring depth and variety in understanding spiritual needs. Third, we did not try to disregard our presupposition during interviews but sought to have a reflective attitude during group discussion in which we let our own pre-understanding be challenged and tried to be open to new ideas by asking follow-up questions to the participants [46,60]. In light of the reflections mentioned, we considered focus groups as a suitable choice of method. All four authors also worked together in the analysis process and made agreement regarding sub theme and main theme. Findings were presented to other independent researchers at seminars and research groups, as well as for nurses and care workers in practice as part of the validation of the themes. Researchers and participants did not know each other before the study. It was therefore important to establish familiarity, confidence and trust in the group initially [38,61]. The participants’ knowledge of the researchers was based on the written information given before the interview. In addition to scientific insight in this research field, the interviewers had a prior understanding from clinical experience in dementia care.

### Limitations

Findings from this study cannot be generalised. Nevertheless, many of the issues and challenges can be recognised in different contexts within dementia care. Norway is considered to be a multi-cultural society to a greater extent than before. Yet the majority of residents in the nursing homes in this study where ethnic Norwegians and well known with the Norwegian church traditions. One limitation is therefore that the nurses had limited experience with different religions. A greater diversity could have expanded the perspectives some more.

## Conclusion

In this study, we found three detailed areas that may explain nurses’ and care workers’ experiences of spiritual needs in residents with dementia. First, nurses' experiences were mostly concentrated on residents’ needs for meaning, as experienced inter-personally, intra-personally, and trans-personally. Second, the nurses desired to create sources of meaning for the residents, in addition to making the resident’s own sources available as much as possible, yet they admitted this effort was difficult. Third, sources of meaning, according to Schnell’s [34] description of people in the general population, can also be applied to people with dementia. However, dementia might limit the resident from retrieving and creating sources of meaning.

The study revealed there is a need to develop more knowledge about residents ‘spiritual needs and how these needs are expressed in people with dementia in nursing homes.

Reed's [27] definition of spirituality (related to meaning making on an inter-personal, intra-personal and trans-personal level), in addition to a more systematic approach consistent with Schnell’s [34] sources of meaning, can be a useful way to recognise and experience residents’ spiritual needs. This method can therefore improve clinical practice and create greater awareness of residents’ spiritual needs in the future.

## Competing interests

The authors declare they have no competing interests.

## Authors’ contributions

Substantial contributions to conception and design: LØ, KK, SH, LJD. Acquisition of data, analysis and interpretation of data: LØ, KK, SH, LJD. Preparing, drafting and critically editing the manuscript: LØ, KK, SH, LJD. All authors read and approved the final manuscript.

## Pre-publication history

The pre-publication history for this paper can be accessed here:

http://www.biomedcentral.com/1472-6955/13/12/prepub

## References

[B1] McSherry W Making sense of spirituality in nursing and health care practice. An interactive approach. Volume 2 edt 2006 London: Jessica Kingsley Publishers

[B2] O'Brien ME Spirituality in nursing: standing on holy ground 2011 Sudbury: Mass.; Jones & Bartlett Learning

[B3] Touhy TA Brown C Smith CJ Spiritual caring: end of life in a nursing home J Gerontol Nurs 2005 31 9 27 35 16190010 10.3928/0098-9134-20050901-07

[B4] Molzahn AE Spirituality in later life: effect on quality of life J Gerontol Nurs 2007 33 1 32 39 17305267 10.3928/00989134-20070101-07

[B5] Beuscher L Beck C A literature review of spirituality in coping with early-stage Alzheimer's disease J Nurs Healthcare of Chronic Illnesses 2008 17 5A 88 97 10.1111/j.1365-2702.2007.02126.x 18298759

[B6] Edvardsson D Winblad B Sandman PO Person-centred care of people with severe Alzheimer's disease: current status and ways forward Lancet Neurol 2008 7 4 362 367 10.1016/S1474-4422(08)70063-2 18339351

[B7] Vance DE Spiritual Activities for Adults with Alzheimer's Disease: The Cognitive Components of Dementia and Religion J Relig Spirituality Aging 2004 17 1–2 109 130

[B8] Powers BA Watson NM Spiritual nurturance and support for nursing home residents with dementia Dementia 2011 10 1 59 80 10.1177/1471301210392980

[B9] Carr TJ Hicks-Moore S Montgomery P What's so big about the 'little things': A phenomenological inquiry into the meaning of spiritual care in dementia Dementia 2011 10 3 399 414 10.1177/1471301211408122

[B10] Ross L Spiritual care in nursing: an overview of the research to date J Clin Nurs 2006 15 7 852 862 10.1111/j.1365-2702.2006.01617.x 16879378

[B11] Pike J Spirituality in nursing: a systematic review of the literature from 2006–10 Br J Nur 2011 20 12 743 749 10.12968/bjon.2011.20.12.743 21727836

[B12] Gaskamp C Sutter R Meraviglia M Adams S Titler MG Evidence-based guideline: Promoting spirituality in the older adult J Gerontol Nurs 2006 32 11 8 13 17112133 10.3928/00989134-20061101-03

[B13] Power J Religious and spiritual care Nurs Older People 2006 18 7 24 27 16927547 10.7748/nop.18.7.24.s15

[B14] Tanyi RA Towards clarification of the meaning of spirituality J Adv Nurs 2002 39 5 500 509 10.1046/j.1365-2648.2002.02315.x 12175360

[B15] Goodall MA The evaluation of spiritual care in a dementia care setting Dementia 2009 8 2 167 183 10.1177/1471301209103249

[B16] Dalby P Sperlinger DJ Boddington S The lived experience of spirituality and dementia in older people living with mild to moderate dementia Dementia 2012 11 1 75 94 10.1177/1471301211416608

[B17] Bursell J Mayers C Spirituality within dementia care: perceptions of health professionals Br J Occupational Therapy 2010 73 4 144 151 10.4276/030802210X12706313443866

[B18] MacKinlay E Trevitt C Living in aged care: Using spiritual reminiscence to enhance meaning in life for those with dementia Int J Mental Health Nurs 2010 19 6 394 401 10.1111/j.1447-0349.2010.00684.x 21054725

[B19] Dwyer LL Nordenfelt L Ternestedt BM Three nursing home residents speak about meaning at the end of life Nursing Ethics 2008 15 1 97 109 10.1177/0969733007083938 18096585

[B20] Bickerstaff KA Grasser CM McCabe B How elderly nursing home residents transcend losses of later life Holist Nursng Pract 2003 17 3 159 165 10.1097/00004650-200305000-00007 12784900

[B21] McEwen M Spiritual nursing care: state of the art Holist Nurs Pract 2005 19 4 161 168 10.1097/00004650-200507000-00007 16006830

[B22] Collin M The search for a higher power among terminally ill people with no previous religion or belief Int J Pall Nurs 2012 18 8 384 389 10.12968/ijpn.2012.18.8.384 23123983

[B23] Narayanasamy A Clissett P Parumal L Thompson D Annasamy S Edge R Responses to the spiritual needs of older people J Adv Nurs 2004 48 1 6 16 10.1111/j.1365-2648.2004.03163.x 15347405

[B24] Weitzel T Robinson S Barnes MR Berry TA Holmes JM Mercer S Foster T Allen L Victor DA Vollmer CM Steinkruger K Friedrich LA Plunkett D Kirkbride GL The Special Needs of the Hospitalized Patient with Dementia MEDSURG Nurs 2011 20 1 13 19 21446290

[B25] Chiu L Emblen JD Van Hofwegen L Sawatzky R Meyerhoff H An Integrative Review of the Concept of Spirituality in the Health Sciences West J Nurs Res 2004 26 4 405 428 10.1177/0193945904263411 15155026

[B26] Nixon AV Narayanasamy A Penny V An investigation into the spiritual needs of neuro-oncology patients from a nurse perspective BMC Nurs 2013 12 2 10.1186/1472-6955-12-2 23374999 PMC3567993

[B27] Reed PG An emerging paradigm for the investigation of spirituality in nursing Res Nurs Health 1992 15 5 349 357 10.1002/nur.4770150505 1529119

[B28] Register ME Herman J Quality of life revisited: the concept of connectedness in older adults Adv Nurs Sci 2010 33 1 53 63 10.1097/ANS.0b013e3181c9e1aa 19996933

[B29] Buck HG Spirituality: concept analysis and model development Holist Nurs Pract 2006 20 6 288 292 10.1097/00004650-200611000-00006 17099417

[B30] McBrien B Spirituality. A concept analysis of spirituality Br J Nurs (BJN) 2006 15 1 42 45 10.12968/bjon.2006.15.1.20309 16415748

[B31] Pesut B Fowler M Taylor EJ Reimer-Kirkham S Sawatzky R Conceptualising spirituality and religion for healthcare J Clin Nurs 2008 17 21 2803 2810 10.1111/j.1365-2702.2008.02344.x 18665876

[B32] Molzahn A Sheilds L Bruce A Stajduhar K Makaroff KS Beuthin R Shermak S People living with serious illness: stories of spirituality J Clin Nurs 2012 21 15/16 2347 2356 22788566 10.1111/j.1365-2702.2012.04196.x

[B33] Koslander T da Silva AB Roxberg A Existential and spiritual needs in mental health care: an ethical and holistic perspective J Holis Nurs 2009 27 1 34 42 10.1177/0898010108323302 19176899

[B34] Schnell T Individual differences in meaning-making: Considering the variety of sources of meaning, their density and diversity Pers Indiv Differ 2011 51 5 667 673 10.1016/j.paid.2011.06.006

[B35] Schnell T The Sources of Meaning and Meaning in Life Questionnaire (SoMe): Relations to demographics and well-being J Pos Psychol 2009 4 6 483 499 10.1080/17439760903271074

[B36] Bradbury-Jones C Sambrook S Irvine F The phenomenological focus group: an oxymoron? J Adv Nurs 2009 65 3 663 671 10.1111/j.1365-2648.2008.04922.x 19222664

[B37] Morgan DL The focus group guidebook. Volume 1 1998 Thousand Oaks, Calif: Sage

[B38] Krueger RA Moderating focus groups. Volume 4 1998 Thousand Oaks, Calif: Sage

[B39] Selbaek G Kirkevold O Engedal K The prevalence of psychiatric symptoms and behavioural disturbances and the use of psychotropic drugs in Norwegian nursing homes Int J Ger Psychiatry 2007 22 9 843 849 10.1002/gps.1749 17193341

[B40] Hummelvoll JK Severinsson E Researchers' experience of co-operative inquiry in acute mental health care J Adv Nurs 2005 52 2 180 188 10.1111/j.1365-2648.2005.03570.x 16164479

[B41] Granerud A Severinsson E Knowledge about social networks and integration: a co-operative research project J Adv Nurs 2007 58 4 348 357 10.1111/j.1365-2648.2007.04239.x 17425599

[B42] Wibeck V Dahlgren MA Oberg G Learning in focus groups: An analytical dimension for enhancing focus group research Qual Res 2007 7 2 249 267 10.1177/1468794107076023

[B43] Lindseth A Norberg A A phenomenological hermeneutical method for researching lived experience Sca J Caring Sci 2004 18 2 145 153 10.1111/j.1471-6712.2004.00258.x 15147477

[B44] Gustafsson G Norberg A Strandberg G Meanings of becoming and being burnout – phenomenological-hermeneutic interpretation of female healthcare personnel's narratives Sca J Caring Sci 2008 22 4 520 528 10.1111/j.1471-6712.2007.00559.x 19000092

[B45] Ricœur P Interpretation theory: discourse and the surplus of meaning 1976 Fort Worth, Tex: Texas Christian University Press

[B46] Gadamer H-G Weinsheimer J Marshall DG Truth and method 2004 London: Continuum

[B47] Narayanasamy A Spiritual care. The puzzle of spirituality for nursing: a guide to practical assessment Br J Nurs (BJN) 2004 13 19 1140 1144 10.12968/bjon.2004.13.19.16322 15573007

[B48] Rykkje L Eriksson K Råholm M-B A qualitative metasynthesis of spirituality from a caring science perspective Int J Hum Caring 2011 15 4 40 53

[B49] Beuscher L Grando VT Using spirituality to cope with early-stage Alzheimer's disease West J Nurs Res 2009 31 5 583 598 10.1177/0193945909332776 19282270 PMC3079522

[B50] Baumeister RF Meanings of life 1991 New York: Guilford Press

[B51] Park CL Paloutzian RF, Park CL Religion and Meaning Handbook of the psychology of religion and spirituality 2005 New York: Guilford Press 295 314

[B52] Park CL Religion as a meaning-making framework in coping with life stress J Soc Issues 2005 61 4 707 729 10.1111/j.1540-4560.2005.00428.x

[B53] MacKinlay EB Trevitt C Spiritual care and ageing in a secular society Med J Aust 2007 186 10 Suppl 21 17516891 10.5694/j.1326-5377.2007.tb01048.x

[B54] Edvardsson D Nordvall K Lost in the present but confident of the past: experiences of being in a psycho-geriatric unit as narrated by persons with dementia J Clin Nurs 2008 17 4 491 498 18205681 10.1111/j.1365-2702.2006.01826.x

[B55] Hodge DR Horvath VE Spiritual Needs in Health Care Settings: A Qualitative Meta-Synthesis of Clients' Perspectives Soc Work 2011 56 4 306 316 10.1093/sw/56.4.306 22308663

[B56] Teeri S Välimäki M Katajisto J Leino-Kilpi H Nurses perceptions of older patients integrity in long-term institutions Sca Jf Caring Sci 2007 21 4 490 499 10.1111/j.1471-6712.2007.00499.x 18036012

[B57] Frankl VE Hammond C Hunt S Batthyany A Man's search for ultimate meaning 2011 London: Rider

[B58] Webb C Kevern J Focus groups as a research method: a critique of some aspects of their use in nursing research J Adv Nurs 2001 33 6 798 805 10.1046/j.1365-2648.2001.01720.x 11298218

[B59] Streubert HJ Carpenter DR Qualitative research in nursing: advancing the humanistic imperative 2011 Philadelphia: Lippincott Williams & Wilkins

[B60] Naden D Hermeneutics and observation–a discussion Nurs Inq 2010 17 1 75 81 10.1111/j.1440-1800.2009.00472.x 20137033

[B61] Morgan DL Focus groups as qualitative research 1997 Thousand Oaks, Calif: Sage Publications

